# Underestimation in Reporting Excess COVID-19 Death Data in Poland during the First Three Pandemic Waves

**DOI:** 10.3390/ijerph19063692

**Published:** 2022-03-20

**Authors:** Marcin Piotr Walkowiak, Dariusz Walkowiak

**Affiliations:** 1 Department of Preventive Medicine, Poznan University of Medical Sciences, 60-356 Poznan, Poland; marcinwalkowiak@ump.edu.pl; 2 Department of Organization and Management in Health Care, Poznan University of Medical Sciences, 60-356 Poznan, Poland

**Keywords:** excess mortality, excess deaths, COVID-19, Poland, public health

## Abstract

The issue whether official Polish COVID-19 death statistics correctly reflect the actual number of deaths is a contentious issue in public discourse and an important policy-wise question in Poland although it has not been the subject of thorough research so far. There had been clearly elevated excess mortality—5100 (death rate of 2.3 per 10,000) during the first wave, 77,500 (21.0 per 10,000) during the second one, and 48,900 (13.5 per 10,000) in the third. This study finds that during the second and the third pandemic wave, our data on excess mortality will match very well the somewhat belatedly officially reported COVID-19 deaths if we assume that only 60% of cases were officially detected. Based on principal component analysis of death timing, except for the age bracket below 40, where COVID-19 deaths calculated on the basis of our model explain 55% of excess mortality, for the remaining age groups, combined COVID-19 deaths explain 95% of excess mortality. Based on the share of excess mortality attributable to COVID-19 during the second wave, this infection in Poland caused the death of 73,300 people and not of 37,600 as officially reported. The third wave caused 46,200 deaths instead of the reported 34,700. The first wave was, indeed, as officially reported, very mild, and the number of excess deaths was too low to be used to calculate COVID-19 deaths directly. However, assuming that the detection rate remained comparable to the average in subsequent waves, we can set the number of deaths at 3500 instead of the reported 2100.

## 1. Introduction

Since the outbreak of the 2019 coronavirus (COVID-19) pandemic, excess mortality has been observed in many countries of the world. The first reports in this regard came in the first half of 2020 immediately after the first wave of the pandemic. Excess mortality in Europe in the aftermath of the coronavirus pandemic was first described by Vestergaard et al. [1], who, in their rapid communication, noted the sharp increase in the number of deaths during this period in March and April 2020. Of course, the problem of excess mortality itself had already been described before in connection with influenza pandemics and heatwaves all over the world [2,3,4,5]. However, it was only during the COVID-19 pandemic that the phenomenon reached unprecedented proportions. In the case of Italy, whose northern regions have been dramatically affected by the effects of the COVID-19 pandemic, with observed shortages of resources [6], it is estimated that there must have been an excess of 47,490 deaths between February and May 2020 alone [7]. In Belgium [8], between 20 March and 28 April, excess deaths are estimated at 7917, while in Portugal, excess deaths estimates between March 1 and April 22 range from 2400 to 4000 deaths [9].

Estimates of excess deaths are not easy to make and involve many assumptions. Moreover, we must be aware that in each case, we are dealing only with a better or worse approximation. Such comparisons are only as good as the estimation method. However, regardless of the method used, in the case of comparisons with other countries, Poland appears in each list of countries with the highest level of excess deaths. Bottcher et al. [10] found Poland among ten countries with the largest numbers of excess deaths in year 2020 and in the first seven weeks of 2021; Islam et al. [11] included it among top five high-income countries as regards the absolute number of excess deaths. In Kelly et al. [12], where 35 countries were studied, Poland ranked first with regard to residual mortality rates. Sanmarchi et al. [13], analysing 67 countries of the world, established that Poland was one of those with the highest number of excess deaths per 100,000 inhabitants; if only OECD members are considered, Poland would rank third. Similar results were obtained by Timonin et al. [14], who, depending on the adopted one of two proposed methodologies, ranked Poland second or eighth in the group of 37 countries in terms of excess deaths.

For the purpose of shaping public health policies, the information on the actual number of additional deaths caused directly or indirectly by the pandemic is crucial. However, official COVID-19 death figures all over the world, for methodological and technical reasons, tend to provide inconsistent information, making it hard to effectively compare data between countries. WHO estimates that in 2020, COVID-19 deaths were globally seriously underestimated, as the official figures combined reached 1.8 million, while excess death statistics implied 3.3 million [15]. For Russia, the official COVID-19 death register explains only 2 out of every 11 excess deaths that occurred during the pandemic [15,16]. The situation is the opposite for England and Wales—based on official estimates, the overall excess death toll from 1 March 2020 to 7 January 2021 was 127,700 [17]. For the same area and period, the number of deaths within 28 days of a positive test was 140,000. Further analysis instead of filtering out cases of merely dying with COVID-19 led to an even greater subsequent inflation of this value since, according to death certificates, COVID-19 was a contributing factor in 157,600 cases [18]. For Italy, official COVID-19 deaths are considered to be both under- and overestimated based on the testing availability during a particular wave [19].

In Poland, the spread of this infection was generally somewhat delayed in relation to Western European countries, which gave the authorities extra time to react, especially during the first wave. As early as mid-March 2020, educational facilities from kindergartens to universities were closed, and further restrictions were ramped up since April: the curfew for minors was introduced, entry to parks was forbidden, and shops were not allowed to serve more than three customers per every cash register. Hotels, restaurants, hairdressers, beauty parlours, and gyms were closed. During summer, most restrictions were lifted. The whole country was divided into colour-coded (green, yellow, and red) zones according to the number of new COVID-19 cases. However, in reaction to dramatically growing infection rates, restrictions were reintroduced in mid-October, including the closing of non-essential shops and of recreational and sport facilities; also, remote learning was reintroduced. These restrictions initially took place only in red zones although before the end of October, the whole country became a red zone. The peak was a sudden ban on traditional visits to cemeteries around All Saints’ Day (1 November). After partially lifting the restrictions in mid-winter, during the third wave, they were mostly reintroduced.

The purpose of this study is to calculate the number of excess deaths during the first, second, and third wave of COVID-19 pandemic in Poland, that is, from 2020-W10 to 2021-W26 (2 March 2020 to 4 July 2021), both disaggregated on provinces and on demographic groups. Based on principal component analysis (PCA) of the second wave’s excess deaths among various sex and age groups, we would be able to establish what share of excess deaths in all demographic groups can be attributed to a single, dominating cause. This percentage would be extrapolated to the remaining period in order to calculate the number of excess deaths caused directly by COVID-19 infection, and this value would be confronted with the official counts of COVID-19 deaths for the purpose of tracing possible under- or overreporting.

## 2. Materials and Methods

### 2.1. Study Design

Variants of quasi-Poisson generalised linear model [20] for calculation of excess death were considered, as those models seem popular. It is clearly highly suitable for the modelling of datasets containing very small samples, which indeed do follow Poisson distribution. However, it required assuming that after normalization through log transformation, the excess deaths during the pandemic should be compensated by dips in other periods, while the more deadly the pandemic, the more reality should diverge from this assumption. This problem is not unsolvable on its own, as those models are refined further to include variables correcting for suspected causes of excess deaths, such as weather conditions [21] or both heat waves and the influenza season [22] (Supplementary Figures S1 and S2).

It was highly desirable to avoid a situation in which, in order to analyse the pattern of excess deaths, one would start from making serious assumptions concerning which pattern exactly those deaths are supposed to follow. Instead, a mixed linear model was preferred, derived from the model suggested by Verbeeck et al. [23]. The modelled number of deaths was high enough for the central limit theorem to be invoked, and instead of assuming Poisson distribution, one should assume normal distribution, which would make the calculation of baseline mortality more reliable. After some adjustments to the original model, the following formula was applied:

$$Y_{i}=a+b\left(T+\frac{t}{52.25}\right)+csin\left(\frac{2\pi t}{52.25}\right)+dcos\left(\frac{2\pi t}{52.25}\right)+\epsilon$$

where: 

*Y_i_*—predicted number of weekly deaths;

*a*—base number of weekly deaths;

*b*—annual change in number of weekly deaths;

*T*—year, t—ISO week of the year;

*c*, *d*—season-dependent component of weekly deaths, created by superimposed sine and cosine function;

*ε*—error of prediction.

The subsequent algorithm for applying this formula was used. A regression model was at first fitted to the dataset. Subsequently, standardised residuals were calculated. The model was then fitted to the dataset again, but this time, observations were weighted by standardised residuals. However, when standardised residuals were above 1, their weight was set to 0. Finally, the previous step was iterated one more time, but residuals from the weighted model instead of the unweighted one were used as new weights in fitting the model for the third time.

While most of the adjustments from the original mixed model had minor impact, there was one crucial difference, which made the model fitting even less sensitive to high excess values. In the original formula, the standardized residuals above 1 were only replaced by 1 divided by their square root. Additionally, in the original model, a variable was suggested for adjusting some unrelated year-to-year changes, while in this model, it was simplified to a trend line. In the original model, the year was divided into 52 weeks, which is generally a very reasonable simplification except that in this particular case, the modelled ISO year 2020 had 53 weeks.

Following the study by Bustos Sierra et al., matching of timing of registered COVID-19 deaths is confronted with the timing of excess deaths through the calculation of Spearman’s *ρ* (rho) [8]. In addition, in order to avoid oversimplifying the assumption that any excess deaths during the COVID-19 pandemic must have been caused—exclusively and directly—by the virus, an additional test was applied. Weekly data on excess deaths, split for genders and age groups, were analysed using PCA with promax rotation. Assuming that there has been a predominant cause of death simultaneously sweeping through the country, those deaths should have had similar timing detectable through PCA. This method would be applicable only to the dataset from the second wave, which was before the vaccine rollout started protecting the most vulnerable demographics. It should filter out unrelated excess deaths that also happen in other years (such as other viral infections) or deaths caused by the pandemic indirectly due to cancellations of planned medical procedures or deaths of despair during lockdowns. This way of counting COVID-19 deaths should yield a somewhat conservative estimate, as serious differences in the timing of infection of people from different demographics or differences in timespan between infection and death would inevitably lead to some undercounting.

The numbering of waves is widely used in Poland, and the Chief Sanitary Inspector uses this classification as well, stating, however, that it is impossible to precisely separate them, as there are diverging infection dynamics in different provinces [24]. For the purpose of this study, the following division was applied. The separation points are the weeks with the lowest numbers of deaths, with each separation point counted as the last week of the previous wave. For each wave to count as one, we assumed that during every week of a wave, there have to be at least 100 excess deaths. Additionally, the relatively less deadly period between the first deaths in Poland attributed to COVID-19 and the beginning of the second wave is referred to as the first wave. On the basis of this classification, for our purposes here, the first wave lasted from 2020-W10 to 2020-W35 (2 March 2020 to 30 August 2020), the second wave from 2020-W36 to 2021-W05 (31 August 2020 to 7 February 2021), and the third wave from 2021-W06 to 2021-W26 (8 February 2021 to 4 July 2021).

Determining the causes of death and assigning the appropriate death codes is crucial information from the health policy point of view. By assigning the appropriate codes, it is possible to monitor, count, observe trends, and compare the number of deaths from specific causes with the data in other countries. During the pandemic, this issue became even more important. Codes of causes of death in Poland are assigned in accordance with the International Statistical Classification of Diseases and Related Health Problems ICD-10. In the coding of deaths related to the COVID-19 coronavirus infection, the following are taken into account: the cause of death (direct, chain-of-events, and underlying), co-occurring conditions, and confirmation of SARS-CoV-2 infection (a positive test result) [25].

For the purpose of a more transparent presentation of the number of deaths, numerical data are rounded to full hundreds as long as at least two significant digits are kept. Validation of the model is done through mixed linear model, while the final estimation through hierarchical mixed linear model is calculated separately for all the analysed demographic groups and summed together before rounding.

Function estimating base death rate was fitted with Python (libraries: Pandas, Statsmodels). GeoDa 1.18.0 was used for generating maps, while PCA and Spearman’s *ρ* was calculated using JASP 0.12.2.

### 2.2. Data Source

While in general, EuroMomo [26] database would be the natural starting point for such an analysis for European countries, Poland is not a EuroMomo partner. In practice, it was not a serious issue since slightly later-reported but very detailed data on weekly deaths split by genders, regions, and age groups are available from Statistics Poland (GUS). We grouped the datasets into ten-year cohorts of the same gender, with the oldest age bracket covering people of 90 years of age or more. As there was generally very low mortality among young people, we created one age group encompassing all people under 40. The general validation of models is based on the data series since 2010, while in order to take into account some more subtle shifts among age groups, the model for calculating excess deaths during the pandemic was scaled on the basis of the period beginning since 2015. The end datapoint used for fitting the models was theoretically 2021-W48, as the intention was to include the most recent data although in practice, the iteratively applied mechanism of disregarding extreme values would either way effectively eliminate from the fitting any observation from mid-autumn 2021 onward.

Population data for calculating the number of deaths per 10,000 people were taken from Statistics Poland. They are available for 1 January and 1 July; thus, for the purpose of calculating death rates during particular waves, for each wave, we used the most up-to-date relevant population count.

Data on the demographics of identified cases of COVID-19 deaths were collected by the Chief Sanitary Inspector and provided by the Polish Ministry of Health [27]. We discarded an insignificant number of incomplete records (missing information on gender or age). The only drawback of this dataset was the lack of the actual dates of death, and they only could be inferred from the date of reporting. For the purpose of this study, where data are split into ISO-weeks, it was assumed that all reporting was delayed by exactly 4 days, which assigns deaths having occurred over the weekend (and thus reported on Monday at the earliest) to the previous ISO-week. Nevertheless, while this assumption is true for a vast majority of cases, it leads to a grave underestimation of the delay in cases of infection waves, leading to paperwork overload or unclear cases that require autopsy.

## 3. Results

### 3.1. Validating the Formula for the Calculation of Excess Deaths

**Figure 1:**
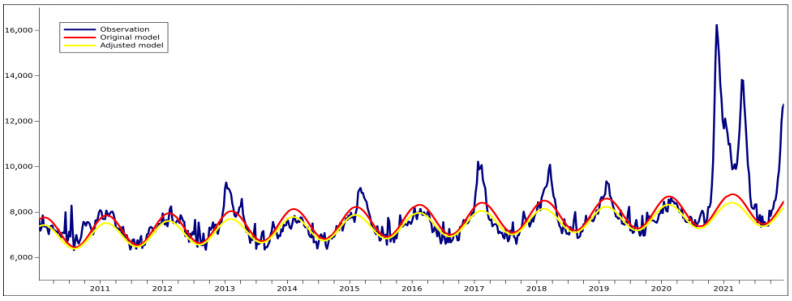
Number of weekly deaths based on reported data, with the explanatory model by Verbeeck et al. and its adjusted version. Dataset from 2010-W01 to 2021-W48; data source: Statistics Poland.

Figure 1 presents the actual weekly death rate since the beginning of 2010 with an estimation of the base death rate calculated using both the original formula from Verbeeck et al. [20] and its adjusted version. Both formulas produce relatively similar results although there are subtle differences favouring the adjusted model. The original formula, while downweighting observations with high excess deaths, still gave them enough weight to calculate the years with a significantly negative number of excess deaths. Such weighted adjustment and one additional fitting did not simply move the axis slightly lower, but it also clearly adjusted it in the desired way, i.e., it kept the lowest values in summer only subtly changed, seriously reduced the base peak in the winter, and moved the peak slightly earlier, around midwinter, away from the usual peak of the influenza season in Poland. A model assuming the low base is necessary especially for calculating COVID-19 deaths in a year with restrictions that should have generally curbed the spread of infectious diseases.

**Figure 2:**
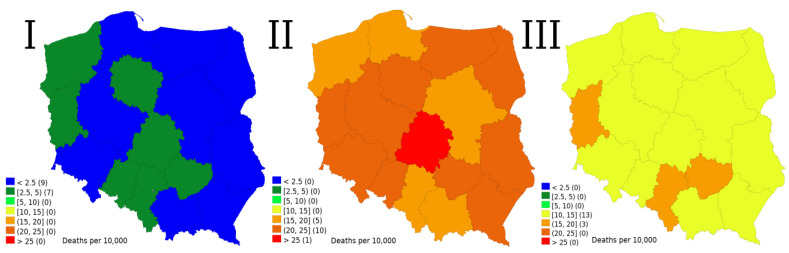
Excess mortality rate during the three waves of COVID-19 pandemic in Poland per 10,000 people; I—1st wave, II—2nd wave, III—3rd wave; data source: Statistics Poland

Figure 2 shows excess mortality rates in Poland per 10,000 inhabitants during the three consecutive pandemic waves. As presented, there was some minor regional variability although the predominant differentiating factor was the period. In Poland, the spread of this infection was generally somewhat delayed in relation to Western European countries. Therefore, during the first wave, there was enough time to react through lockdowns and voluntary behavioural changes, which led to a relatively low death rate of 2.3 per 10,000. The highest number of deaths occurred during the second wave—21.0 per 10,000. During the third wave, vaccination rollout started, which reduced the number of excess deaths to 13.5 per 10,000. It was also during the third wave that the highest number of infections was observed, both based on official count and on the inferred level from excess mortality in unvaccinated age brackets.

### 3.2. Relation between Excess Deaths and COVID-19 Deaths

**Figure 3:**
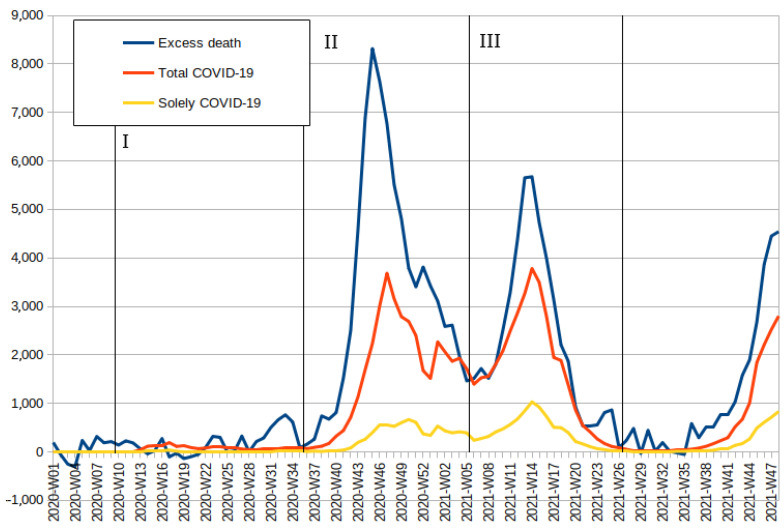
Relation between weekly excess mortality and weekly officially recorded deaths for which COVID-19 was sole or among the listed causes of death, shifted back 4 days to adjust for reporting delay. Data source: Statistics Poland, Polish Chief Sanitary Inspector.

Figure 3 illustrates the relation between excess deaths, deaths classified as having COVID-19 among their causes, and those where it was the sole recorded cause. In 2020, both before the beginning and during the first wave, there are even periods with a slightly negative number of excess deaths. The peak at the end of the first wave actually coincides with a heat wave [28], while Spearman’s *ρ* between the total COVID-19 death count and the number of excess deaths remains not only statistically insignificant even for *p* < 0.1 but, moreover, negative. From the onset of the second wave, all the analysed death metrics increase sharply and become highly correlated. For the second wave, Spearman’s *ρ* increases to 0.759 (95% confidence interval—(CI) 0.505–0.892, with *p* < 0.001), while for the third one, it rises further to 0.958 (CI 0.899–0.893, *p* < 0.001), which implies a strong relationship during subsequent waves. According to official COVID-19 death data, the second and third waves were comparable in size although excess deaths clearly show that the second wave was much worse. While nominally, those data imply that during the second wave, approximately every second COVID-19 death was missed, and during the third one, every third death was missed, the situation seems to be more complicated. During the low between those two waves, the number of COVID-19 cases was approximately equal to the number of excess deaths, which is most likely to have been simply delayed reporting from the second wave. Therefore, while both lower discrepancy in the overall number of deaths and increased correlation suggest a clear improvement in reporting, the cases from those two periods are seriously intermingled; thus, for the calculation of the relationship between the number of excess deaths and the officially counted COVID-19 deaths, both waves have to be combined together.

Based on modified methodology, the number of excess deaths in each analysed demographic group was calculated for the whole period of the second and third waves combined, and it is presented in Table 1. For the dataset before the mass vaccine rollout, it was possible to calculate the PCA loadings matrix to verify whether the relationship between excess deaths remains constant in all demographic groups in a way that would imply the existence of one dominating factor: in this case, COVID-19 infection waves. The squares of PCA component loadings are presented in Table 1. Based on that, inferred COVID-19 deaths are calculated on the basis of our model. While there is a very strong underlying pattern, the relationship is not straightforward in the younger age brackets, especially among people under 40, among whom it is responsible for 58% of excess deaths for men and 48% for women. The relationship seems actually strongest for people in their 70s and 80s, while it slightly declines for those who are at least 90.

Overall, the calculated detection rate of deaths caused by COVID-19 is 60%. Assuming that the same detection probability existed during the first wave, it would mean that instead of 2100 people, as many as 3500 died, while the excess deaths for this period amounted to 5700. Different assumptions concerning this estimate would not change the overall picture, as during the second wave, 73,300 (instead of 37,600) people died of COVID-19 and during the third wave, 46,200 people (instead of 34,700).

## 4. Discussion

The results calculated on the basis of the model show that the number of excess deaths was much higher than official COVID-19 death reports indicated although interpretation of the value we obtained would require some additional context. Based on the model presented in Figure 1, the average number of excess deaths in the years 2010–2019 was 9000 +/− 6300. During 70 weeks of the three waves, there have been 8400 excess deaths that, based on the model, are not attributable directly to COVID-19. The method of attributing deaths to COVID-19 assumes a perfectly uniform timing of deaths in all demographic groups, which is highly unlikely, and each percentage of underestimation should reduce the number of deaths requiring other explanation by 1200. In 2020, not only the number of detected cases of influenza and influenza-like illnesses was clearly lower than average (3.1 million of cases as opposed to 4.6 million +/− 0.57 in the 5 preceding years), but the number of hospitalisations due to this reason was lower as well—15,300 (in previous 5 years: 21,500 +/− 4500) [24]. Since the number of diagnosed cases was not only 2.6 standard deviations (SD) lower than the norm (which on its own could have been mostly just a diagnosis issue of people foregoing medical treatment for minor infections), but the number of hospitalisations also went down by 1.3 SD, it should rather imply a lower number of other infections. While from the severity of lockdowns and cancelled planned medical procedures, one expects additional, excess deaths indirectly caused by COVID-19, the remaining excess death number looks mundane and well within the value that could be explained away by other factors. Moreover, even though the situation in the healthcare system was perceived by the public as quite bad, excess deaths cannot be explained by its collapse—the relation between reported COVID-19 deaths and excess death remains roughly linear, while a system on the brink of collapse should have created a clearly nonlinear relationship. Thus, either the indirect harm at least in the short run was indeed minuscule, or—which is more likely—restrictions led to a decrease in the number of other infections, which reduced overall mortality enough to mask on aggregated numbers some relatively small number of indirect deaths caused by the pandemic.

Regardless of the exact figure, our results confirm—despite some suspected discrepancies between the overall official COVID-19 statistics and our estimates—the government’s success in containing the first wave in Poland, as indicated by low numbers of excess deaths. Thus, the initial concern about the shortage of polymerase chain reaction tests at the time of the first wave, leading to high positive rates among tested cases and possibly masking the true scope of the pandemic, turned out to be exaggerated. Later, unfortunately, that was not the case, as the number of excess deaths dramatically increased during the second wave, with at least 95% of them caused directly by COVID-19, with the percentage unlikely to subside during the third wave. The conclusion that excess deaths were due almost exclusively to COVID-19 infections, while indirect COVID-19 pandemic deaths were minuscule, is consistent with the results obtained by Wollschläger et al. [29], who based their study on the timing and spacial analysis of a German dataset. The weak relationship between excess deaths and COVID-19 waves among demographics below 40 is not surprising since not only was there relatively low mortality in this age bracket, but, especially for men, there was also a high share of deaths caused by external factors, such as car accidents, whose number actually tends to go down during lockdowns [30].

Enormous differences between the first and the second wave are in agreement with Bogos et al. [31] and actually are the dominating pattern among former communist EU members. In contrast with the study by Golinelli et al. [32], who analysed the pattern in Lombardy during the first and second wave, in Poland, there was no obvious regional pattern, with the most severely hit regions becoming less vulnerable in the subsequent wave although it could be caused simply by a different level of analysis.

COVID-19 cases in Poland were not only underreported, but they were also bringing the wrong message. There was serious general distrust in the establishment, leading to vaccine hesitancy, especially among socially disadvantaged groups [33]. At the same time, even according to official government COVID-19 death statistics, there was no clear difference in the number of COVID-19 deaths before and after the vaccine rollout. A rudimentary glance at the graphs presenting official COVID-19 deaths was showing to the general public that there was only one policy that did work—continual lockdowns—but it was unattainable in the long run, while starting to vaccinate the most vulnerable segment of society did not bring any visible improvement to the overall death rate. As trust in the establishment was low in general, instead of using its whole authority to explain how safe and effective vaccines are, the government could have relied on lack of trust as a factor acting in its favour. Paradoxically, revealing early in the pandemic the true number of excess deaths and admitting that the truth was much worse than originally disclosed to the public and that the attempts to contain the virus were failing to deliver the expected goals might have contributed towards greater acceptance of vaccines. In that narrative, citizens should not expect that the situation be contained soon, and regardless of their concerns about vaccine safety, side effects, and long-term effectiveness against symptomatic disease, they should pursue vaccination, because in the future, that will be their only effective protection.

A more candid approach could have been slightly more convincing for the general public than a deputy health minister reiterating that vaccines [34,35] are really safe because in the government database, there are only 17,700 cases of COVID-19 vaccine side effects, with 14,800 of them mild, such as a sore arm after the injection [36]. Taken at face value, this would be an impressive result after administering over 50 million doses of COVID-19 vaccines. It would mean that even if we include a sore arm, the ratio of side effects is lower than 0.035% of vaccine doses administered, and a single patient is highly unlikely not only to have experienced any but even to personally know someone who, for one day, felt somewhat ill as a result of COVID-19 vaccination. Unfortunately, based on data derived from an official study used to approve the Pfizer vaccine, local reactions were noticed in 84.7% of cases, while systemic reactions were present in 77.4% [37]. Using this particular register to reassure vaccine-hesitant people that side effects are negligible and well monitored by the government is somewhat self-defeating. In order to achieve high vaccination rates, the government would have to either use compulsion or more cogent arguments. As the Polish government considered forcing the unconvinced by introducing vaccine mandates to be too damaging to its popularity [38], it needed a simple and powerful argument. Missing the argument about underestimating the true scale of the pandemic and about vaccination rollout as the factor that actually reduced the third wave was specially harmful in the long run during the fourth wave of the pandemic.

## 5. Conclusions

Our model clearly confirms that in Poland, the first wave of COVID-19 was well contained as officially reported and hardly exerted any directly visible impact on excess mortality. In the second and the third wave, however, COVID-19 deaths were seriously underestimated in the official statistics, constituting only 60% of the actual cases, as calculated on the basis of our model. Moreover, government data inaccurately presented those two waves as comparable in size, while excess mortality was clearly much higher during the second wave than in the third one. When we matched the timing between excess deaths among different demographic groups and between the deaths officially recorded as due to COVID-19 and the deaths in general, we found that at least 95% of excess deaths must have been directly caused by COVID-19, thus leaving very little room for other possible causes.

## 6. Limitations

The main limitations of this study are implicit assumptions of modelling excess deaths in general—we assume that weekly mortality in general follows annual sine-like function, interrupted by sudden spikes. This leads to a situation where an infectious disease that kills in short bursts of epidemic would cause excess deaths, while the same pathogen causing the same number of deaths slowly throughout the whole winter would merely increase base level. This fact affected the calculation indirectly through changing the base level above which deaths were considered as excessive. In this particular case, where the number of excess deaths was extraordinarily high, it had limited impact.

## Figures and Tables

**Table 1:** Comparison of excess mortality, COVID-19 deaths calculated on the basis of our model, and officially reported COVID-19 deaths by demographic groups. Data source—officially reported deaths: Statistics Poland, Polish Chief Sanitary Inspector, inferred deaths: own calculation.

Demographics	Excess Mortality	PCA Squared	Inferred COVID-19 Deaths	Officially Reported COVID-19 Deaths	Detection Rate
M0_39	1400	58%	830	450	54%
M40_49	2800	85%	2400	1000	43%
M50_59	6300	94%	5900	2700	45%
M60_69	16,400	97%	15,800	10,000	63%
M70_79	20,900	98%	20,400	13,500	66%
M80_89	17,400	96%	16,800	10,600	63%
M90plus	4500	86%	3900	2700	70%
F0_39	730	48%	350	230	66%
F40_49	1200	74%	920	450	49%
F50_59	2700	89%	2400	1300	54%
F60_69	8600	95%	8200	5000	62%
F70_79	14,900	97%	14,400	8800	61%
F80_89	20,200	97%	19,600	11,100	57%
F90plus	8400	92%	7700	4500	58%
Total	126,400	95%	119,600	72,300	60%

## Data Availability

https://www.gov.pl/web/gis/stan-sanitarny-kraju-w-2020-roku (accessed on 13 March 2022); https://www.meteo.waw.pl/hist.pl (accessed on 13 March 2022); https://basiw.mz.gov.pl/index.html#/visualization?id=3653 (accessed on 13 March 2022).

## Supplementary Materials

The following supporting information can be downloaded at: https://www.mdpi.com/article/10.3390/ijerph19063692/s1, Figure S1: Quasi-Poisson generalised linear models fitted to Polish death data, including COVID-19 pandemic period; Figure S2: Fitting quasi-Poisson generalised linear models fitted to Polish death data before COVID-19 pandemic compared with adjusted model.

## Abbreviations

COVID-19	the coronavirus disease 2019
SD	standard deviation
PCA	principal component analysis
