# Supplementary material for: Underestimation in Reporting Excess COVID-19 Death Data in Poland during the First Three Pandemic Waves
Source: Int J Environ Res Public Health. 2022 Mar 20;19(6):3692. doi: 10.3390/ijerph19063692 (PMC8954142; doi:10.3390/ijerph19063692)
Supplement: Supplementary file 1 [file ijerph-19-03692-s001.zip › ijerph-1608197-supplementary.pdf]

Supplementary materials

Standard quasi-Poisson generalised linear model was a reasonable simplification in the case of a moderate number of excess deaths due to factors such as the annual flu season or a heat wave. In the case of trying to fit it directly to longer periods of elevated mortality, it starts fitting periods of elevated mortality to model as if they were part of normal background. We presented in Figure S1 two such models: one simpler with just a trend line, and another, normally considered more sophisticated as it includes as an extra variable the average number of deaths from the last five years [20]

Figure S1: Quasi-Poisson generalised linear models fitted to Polish death data including COVID-19 pandemic period

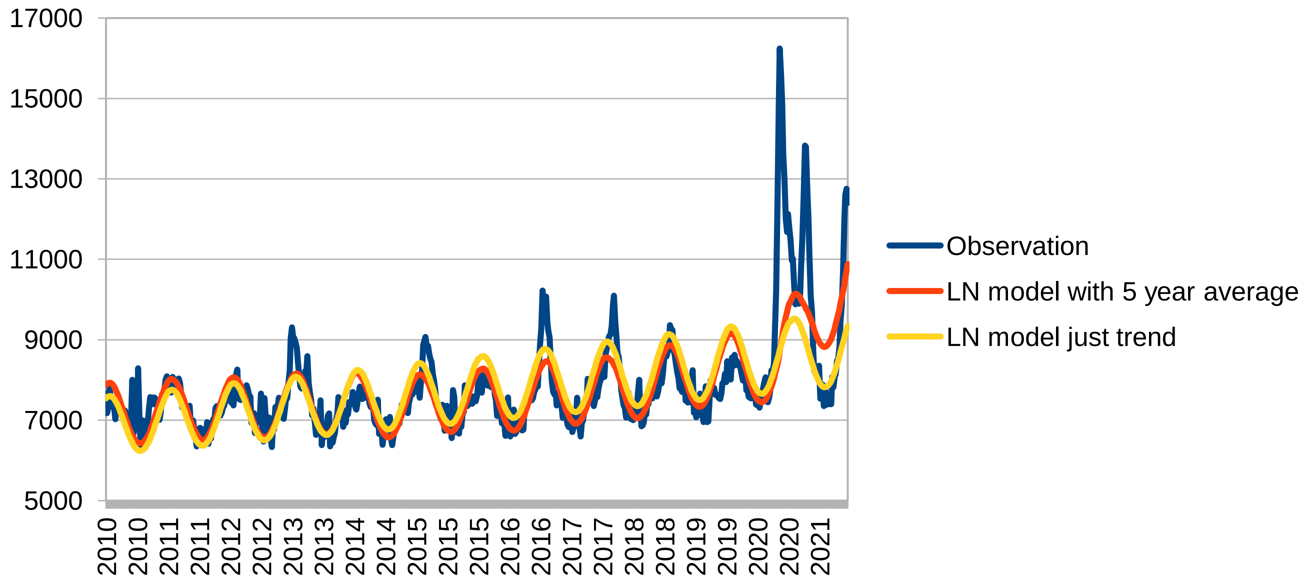

As presented in Figure S2, it was possible to reasonably fit in a simple quasi-Poisson generalised linear model to pre-COVID-19 dataset and subsequently to extrapolate it. The result was not dramatically different from the actually applied model, but there were some clear differences. This alternative model was clearly estimating a somewhat higher base mortality rate and implying the existence of multiple winter seasons with markedly negative excess mortality, which was a clear drawback of such model.

Figure S2: Fitting quasi-Poisson generalised linear models fitted to Polish death data before COVID-19 pandemic compared with adjusted model

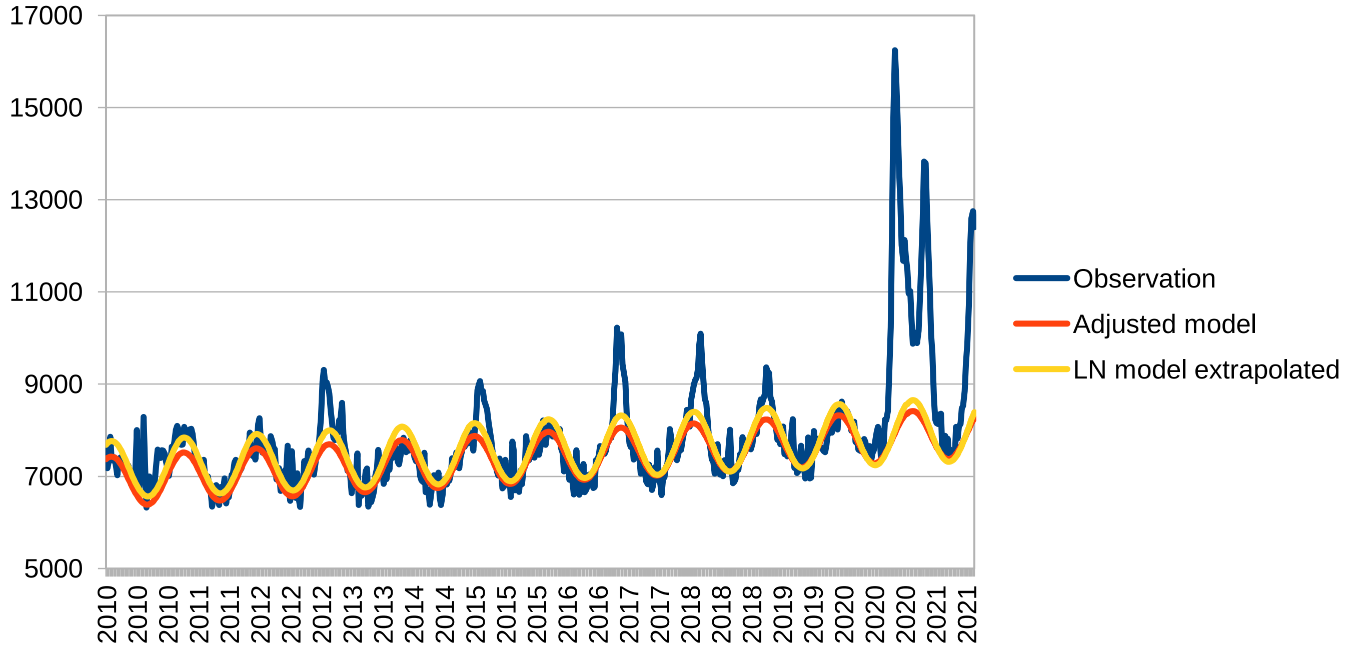

References:

20. Barnard, S.; Chiavenna, C.; Fox, S.; Charlett, A.; Waller, Z.; Andrews, N.; Goldblatt, P.; Burton, P.; De Angelis, D. Methods for Modelling Excess Mortality across England during the COVID-19 Pandemic. *Stat Methods Med Res* **2021**, 9622802211046384, doi:10.1177/09622802211046384.
